# Laponite-Modified Biopolymers as a Conformable Substrate for Optoelectronic Devices

**DOI:** 10.1021/acsomega.4c03463

**Published:** 2024-07-09

**Authors:** Bruno S. D. Onishi, Rafael S. Carvalho, Ricardo Bortoletto-Santos, Silvia H. Santagneli, Arthur R. J. Barreto, Aline M. Santos, Marco Cremona, Omar G. Pandoli, Mario N. B. Junior, Thales A. Faraco, Hernane S. Barud, Renan L. de Farias, Sidney J. L. Ribeiro, Cristiano Legnani

**Affiliations:** † Institute of Chemistry, São Paulo State University (UNESP), Araraquara, SP 14800-060, Brazil; ‡ Departamento de Física, Pontifícia Univ. Católica do Rio de Janeiro (PUC-Rio), Rio de Janeiro 22451-900, Brazil; § Postgraduate Program in Environmental Technology, University of Ribeirão Preto (UNAERP), Ribeirão Preto 14096-900, Brazil; ∥ Departamento de Química, Pontifícia Univ. Católica do Rio de Janeiro (PUC-Rio), Rio de Janeiro 22451-900, Brazil; ⊥ Departamento de Engenharia Química e de Materiais, Pontifícia Univ. Católica do Rio de Janeiro (PUC-Rio), Rio de Janeiro 22451-900, Brazil; # Departamento de Física, Laboratório de Eletrônica Orgânica (LEO), Univ. Federal de Juiz de Fora (UFJF), Juiz de Fora 36036-330, Brazil; ∇ Laboratório de biopolímeros e Biomateriais (BIOPOLMAt), Univ. de Araraquara (UNIARA), Araraquara 14801-340, Brazil

## Abstract

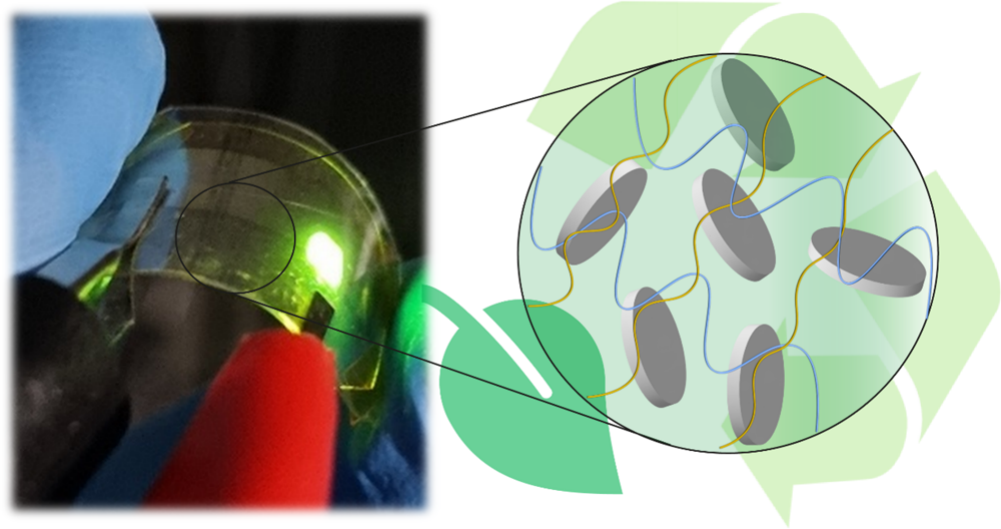

Biopolymers such as carboxymethyl cellulose and hyaluronic acid are alternative substrates for conformable organic light-emitting diodes (OLEDs). However, drawbacks such as mechanical stress susceptibility can hinder the device’s performance under stretched conditions. To overcome these limitations, herein, we developed a nanocomposite based on CMC/HA (carboxymethyl cellulose/hyaluronic acid) and synthetic Laponite, intending to improve the mechanical strength without compromising the film flexibility and transparency (transmittance >80%; 380–700 nm) as substrates for conformable OLEDs. From XRD, FTIR, CP-MAS NMR, and TGA/DTG characterization techniques, it was possible to conclude the presence of Laponite randomly dispersed between the polymer chains. CMC/HA with 5% (w/w) Laponite, CMC/HA 5, presented a higher tensile strength (370.6 MPa) and comparable Young’s modulus (51.0 ± 1.2 MPa) in comparison to the nanocomposites and pristine films, indicating a better candidate for the device’s substrates. To produce the OLED, the multilayer structure ITO/MoO_3_/NPB/TCTA:Ir(ppy)_3_/TPBi:Ir(ppy)_3_/BPhen/LiF was deposited onto the CMC/HA 5 substrate. The OLEDs fabricated using CMC/HA 5 substrates showed higher luminance (12 kcd/m^2^) and irradiance (0.9 mW/cm^2^) values when compared with those based on commercial bacterial cellulose. However, the same device presented a lower efficiency (3.2 cd/A) due to a higher current density. Moreover, the OLED fabricated onto the Laponite-modified biopolymer presented reproducible behavior when submitted to continuous bending stress. Thus, CMC/HA 5 demonstrates potential as a transparent conductor substrate for biopolymer-based OLEDs with comparable performance to commercial bacterial cellulose features.

## 1 Introduction

Biopolymers have emerged as promising alternatives to conventional polymers, characterized by their eco-friendly nature, lighter weight, biocompatibility, and cost-effectiveness.^1−3^ Carboxymethyl cellulose (CMC) is a water-soluble carboxymethylated derivative composed of glucose units bound to β-1,4-glycosidic linkages with some hydroxyls replaced by anionic carboxymethyl groups.^4,5^ Hyaluronic acid (HA) is found as a significant component in the extracellular matrix of animal connective tissues, having hydration and structural stabilization roles. HA comprises glycosaminoglycan polysaccharides with repeating β-1,4-d-glucuronic and β-1,3-*N*-acetyl-d-glucosamine units.^6,7^

Both HA and CMC biopolymers have excellent film-forming properties to produce highly transparent films for multifunctional applications such as textile, food, cosmetic, and pharmaceutical.^5,8−10^ Free-standing CMC and HA-based films can be good candidates as substrates for OLEDs (organic light-emitting diodes), OFETs (organic field effect transistors), and OTFTs (organic thin film transistors).^11^ Additionally, their low-cost manufacturing over glass-based devices has been considered to be an advancement. However, while these organic-based substrates offer benefits over the glass ones in terms of conformability, both HA and CMC biopolymer films have shown some drawbacks that should be taken into consideration, such as mechanical stress susceptibility, more sensitivity to degradation by oxygen, and lower thermal stability than inorganic substrates, which can lead to reduced device performance over time or device failure.

One possibility for overcoming these limitations is the development of a polymeric composite. CMC/HA composites were studied by Kim et al., which related the CMC content to the enhancement of the composite mechanical strength, improving the rigidity and remaining flexibility of the film.^12^ Another possible strategy involves clay mineral incorporation in the film structure, giving rise to the clay–polymer nanocomposites with improved mechanical strength and flexibility preservation.^13,14^ Laponite is a synthetic clay with the empirical formula Na_0.7_^+^[(Si_8_Mg_5.5_Li_0.3_)O_20_(OH)_4_]_0.7_^–^ formed by tetrahedral silicate layers compressing an octahedral layer of magnesium oxide.^15−17^ The octahedral center is coordinated by oxygen anions and hydroxyls and undergoes isomorphic substitution of Mg^2+^ by Li^+^, leading to negative charges on the surface between the layers counterbalanced by Na^+^ cations.^15−19^

Properties such as mechanical strength, water permeability, and transparency of nanocomposite films based on Laponite have been investigated by some researchers. Oliveira et al. have developed a nanocomposite based on CMC-Laponite, finding an enhancement in mechanical strength and a decrease in water permeability with Laponite content.^11^ Interestingly, the synthetic clay did not affect the film transparency. Similar outcomes were found by Perotti et al., which combined bacterial cellulose (BC) with Laponite to produce a film with high tensile strength.^18^ Both studies associated the H-bonding interactions between clay and polymer with mechanical strength results and highlighted the possible application of substrates as conformable insulators for OLEDs.

In the past decades, the OLED technology has been growing with research interest in both industrial and academic research areas due to its characteristics such as low energy consumption, wide viewing angles, high efficiency, color purity, and mechanical flexibility.^20−22^ So far, high efficiency, brightness, and stability under operation have been well solved for commercial devices. However, as pointed out by Murawski and Gather,^23^ besides the displays in optoelectronic devices (TVs, computers, and mobile devices), OLEDs have been extensively studied for biomedical purposes, such as wearable sensors for heart rate and oxygen content in blood measurement, due to the non-invasive and biocompatibility features. Photodynamic therapy and optogenetics also were demonstrated as other possibilities of OLED technology for biomedicine. Savvatev’ev et al.^24^ also reported an application as an integrated OLED-chemical sensor. The authors of these examples highlighted the low temperature and low voltage required for operation, since it works in physiological temperatures of 36–39 °C, and high temperature could damage cells or analytes in the case of integrated sensors. Thus, OLEDs based on biopolymer substrates are desirable for the advancement in this field.

As an integral part of our continuous research on biopolymer-based substrates for optoelectronic devices, this study represents a significant advancement, as we successfully developed a novel and conformable CMC/HA-Laponite composite. The primary objective was to reinforce the substrate while retaining its exceptional transparency and mechanical flexibility. This work not only involves the development and meticulous characterization of CMC/HA-Laponite nanocomposites as substrates for OLEDs but also serves as a compelling proof of concept for the performance of our developed OLED device in comparison to that of the OLED based on commercial BC.

## 2 Experimental Section

### 2.1 Materials

Hyaluronic acid sodium salt (HA) and lithium fluoride (LiF) were purchased from Sigma-Aldrich. Carboxymethyl cellulose sodium salt (CMC) with M.W. = 90,000 (DS = 0.7) was purchased from Acros Organics. Synthetic Laponite RD was kindly supplied by Buntech (Brazil). Silicon dioxide (SiO_2_) and indium tin oxide (ITO) sputtering targets and aluminum (Al) pellets were purchased from Kurt J. Lesker Company. Molybdenum(VI) oxide (MoO_3_), *N*,*N*′-bis(naphthalen-2-yl)-*N*,*N*′-bis(phenyl)-benzidine (β-NPB), 4,4′,4-tris(carbazol-9-yl)triphenylamine (TCTA), 1,3,5-tris(1-phenyl-1*H*-benzimidazol-2-yl)benzene (TPBi), tris(2-phenylpyridine)iridium(III) (Ir(ppy)_3_), and 4,7-diphenyl-1,10-phenanthroline (BPhen) were purchased from Luminescence Technology Corp. All of the materials were used as received.

### 2.2 Composite Film Preparation

The nanocomposite films (conformable substrates) were produced by using the solvent casting methodology. HA and CMC powders were mixed 50% (weight/volume = wt/v) in a proportion of deionized water under magnetic stirring agitation at room temperature (298 K) for 24 h. Laponite was dispersed in 10 mL of deionized water and sonicated (40 kHz, 154 W) for 10 min. The percentages of lamellar material dispersed in the CMC/HA mixture were 2.5, 5, and 20% (weight/weight = wt/wt). After 24 h of continuous agitation, the polymer/clay mixtures were transferred to polystyrene Petri dishes (60 mm × 15 mm) and dried at 30 °C in an air circulation oven for 48 h. For the comparison and interpretation of the structure interactions, the individual pristine films were also prepared according to Table 1.

**Table 1:** Code and Composition of Each Treatment

**code**	**Laponite (g)**	**CMC (g)**	**HA (g)**
CMC		0.50	
HA			0.50
CMC/HA		0.50	0.50
CMC/HA 2.5	0.025	0.50	0.50
CMC/HA 5	0.050	0.50	0.50
CMC/HA 20	0.20	0.50	0.50

The nanocomposite films were kept in this air circulation oven just until they were transferred in the vacuum chamber for the fabrication of the OLED device.

### 2.3 Characterization

Pristine and all prepared nanocomposites were characterized by UV–vis transmittance (300–800 nm), attenuated total reflectance Fourier-transform infrared spectroscopy (ATR-FTIR), X-ray diffraction (XRD), thermogravimetric analysis (TGA), solid state cross-polarization–magic angle spinning nuclear magnetic resonance (CP-MAS NMR), and mechanical properties.

Transmittance was carried out using a Cary 5000 UV–Vis–NIR spectrophotometer (Varian, USA) in the 300–800 nm range. ATR-FTIR spectra (400–4000 cm^–1^) were collected with a NICOLET IS5 Infrared Spectrometer Thermo Scientific, employing attenuated total reflection at the iD3 module with a germanium crystal, at a resolution of 2 cm^–1^ using 32 scans.

XRD patterns were obtained with a Shimadzu diffractometer/LabX XDR-6000 operating at 30 kV and 30 mA. Scans were performed in the 2θ range between 4° and 70° with a scan step of 0.02 and a Cu Kα radiation source (λ = 1.54 Å). The samples were placed in the cavity of the glass sample holder (plate, 25 mm in diameter and 1 mm deep).

TGA ranged from 30 to 800 °C with 8 mg of each sample at a heating rate of 10 °C/min and synthetic air of 60 mL/min (equipment TGA-Q500/TA Instruments).

CP-MAS NMR experiments of the ^29^Si and ^13^C nuclei were conducted in a Bruker Avance III HD 400WB spectrometer (9.4 T) using a double resonance probe of 4 mm. The experiments were conducted at a spinning speed of 10 kHz. The CP-MAS ^29^Si{^1^H} and ^13^C{^1^H} spectra were recorded by using contact times of 4.0 and 3.5 ms and a relaxation delay of 3 s, respectively. All spectra were acquired with TPPM proton decoupling during data acquisition, and chemical shifts are reported relative to the TMS referencing standard.

Mechanical properties on the films were achieved using a DL-2000 (Emic, Brazil) universal testing machine, setting with a 50 kgf load cell, an initial grip separation of 10 mm, and a crosshead speed of 0.83 mm/s.^25^ Mechanical parameters, such as ultimate tensile strengths and elongation at fracture, were collected with Tesc version 3.04 software from the stress–strain curves. Likewise, Young’s modulus was calculated as the slope of the initial linear portion of those curves.

### 2.4 OLED Preparation

To validate the potential of CMC/HA 5 as a conformable substrate for the development of the OLED technology, we fabricated a multilayer device based on a phosphorescent compound. Additionally, we fabricated the same OLED using a commercially available BC substrate, which is a biopolymer platform reference.^26^ This BC substrate, derived from bacterial cellulose, served as the control group in our study, providing a benchmark for comparison and evaluation of the OLED’s performance and characteristics.

A 200 nm SiO_2_ interlayer was deposited onto both polymer substrates to functionalize them, followed by a 150 nm ITO^27^ film used to make the substrates conductive. Both SiO_2_ and ITO thin films were deposited by the rf-magnetron sputtering technique in an argon atmosphere. The SiO_2_ film was deposited at 100 W power for a period of 90 min. On the other hand, the ITO film was deposited using 120 W power for 15 min. An argon flow rate of 30 sccm was maintained during both deposition processes.

The OLED devices were fabricated with the following structure: ITO (150 nm)/MoO_3_ (2 nm)/NPB (30 nm)/TCTA:Ir(ppy)_3_ (7 wt %; 20 nm)/TPBi:Ir(ppy)_3_ (7 wt %; 10 nm)/BPhen (40 nm)/LiF (0.1 nm)/Al (100 nm) (Figure S1). The organic molecules, MoO_3_, and LiF were deposited by the thermal evaporation technique in a high vacuum environment (∼10^–4^ Pa). The deposition system used for the fabrication of the devices is fully integrated within an MBraun glovebox to prevent exposure of materials and devices to the ambient atmosphere. The deposition rate for the organic layers was 0.5 Å/s, and the active area of the devices was about 3 mm^2^. All measurements were performed in an ambient atmosphere and under ambient atmospheric conditions without any encapsulation. The same multilayer device was also fabricated onto a commercial glass/ITO-patterned substrate from Luminescence Technology Corp. as a reference OLED.^28^

## 3 Results and Discussion

### 3.1 Optical and Structural Characterization

**Figure 1:**
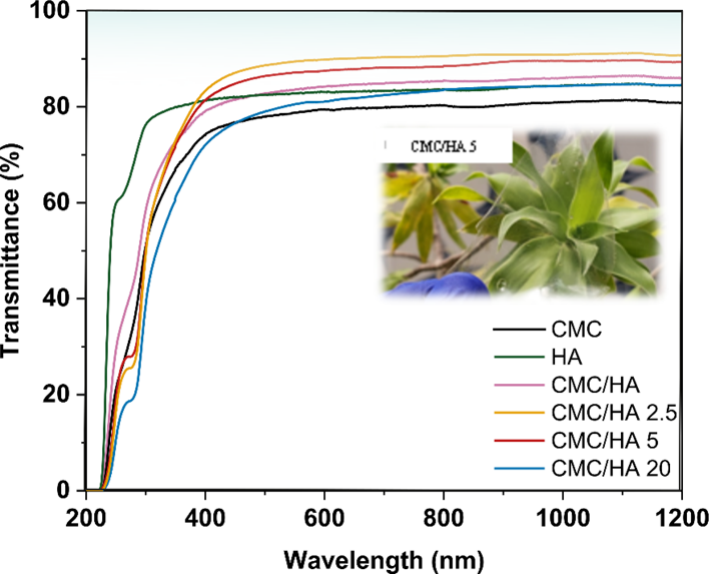
Optical transmittance of the CMC, HA, and nanocomposite films. Inset: picture of the nanocomposite CMC/HA 5 transparent film (photograph taken by the authors).

The prepared nanocomposite films were free-standing, transparent, and macroscopically homogeneous, as shown in Figure 1, which also reveals the good flexibility of the film without breaking or causing damage to the material. The average thickness of nanocomposite films was (100 ± 40) μm. The UV–vis transmittance spectra are also represented in Figure 1, where it is possible to notice the high transmittance in the visible range (380–700 nm) of the substrates with low content of Laponite, 90% for CMC/HA 2.5 and 87% for CMC/HA 5. On the other hand, the Laponite-free and CMC/HA 20 films presented 80% transmittance. Silva et al. suggest an aggregation of cellulose nanofibers promoted by Laponite, resulting in a reduction of light scattering.^29^ The hypothesis could work for the low content of Laponite in the CMC/HA films. However, a higher amount of Laponite could induce some aggregation of the layers of the clay itself.^30^

ATR-FTIR spectra of the CMC/HA nanocomposite films (Figure S2) revealed a combination of the bond vibrations of CMC or HA and Laponite backbones. The broad band at 3359 cm^–1^ corresponds to the OH stretching vibration from CMC and HA and can be assigned to the lattice hydroxyl groups.^31,32^ The bands at 2924 and 2848 cm^–1^ are assigned to symmetric and asymmetric stretching of −CH_2_ groups from CMC and HA structures, while the 1603 cm^–1^ band comes from the −OH bending from water molecules.^33,34^ The bending vibration of the −CH_2_ group appears at 1419, 1375, and 1323 cm^–1^, related to symmetric deformation (also known as crystallinity band), antisymmetric deformation, and wagging motion or out-of-plane deformation, respectively.^29,31,33,35^ The bands at 1159 and 891 cm^–1^ correspond to the stretching of the C–O–C β-glycosidic linkage between the glucose units of CMC and HA, and the band corresponds at 1046 cm^–1^ to the C–O stretching vibration.^31,35^ The band at 999 cm^–1^ is assigned to the Si–O stretching vibration of Laponite tetrahedral layers.^32,36^ When the Laponite content is low, at 2.5 and 5%, this band appears as a shoulder of the CO stretching band. However, with 20% clay mineral, the intensity is enhanced. The 652 cm^–1^ band is attributed to the Mg–OH–Mg bending vibration.^32,36^ From the analysis of the ATR-FTIR spectra, it was possible to verify the successful incorporation of Laponite in the nanocomposites.

**Figure 2:**
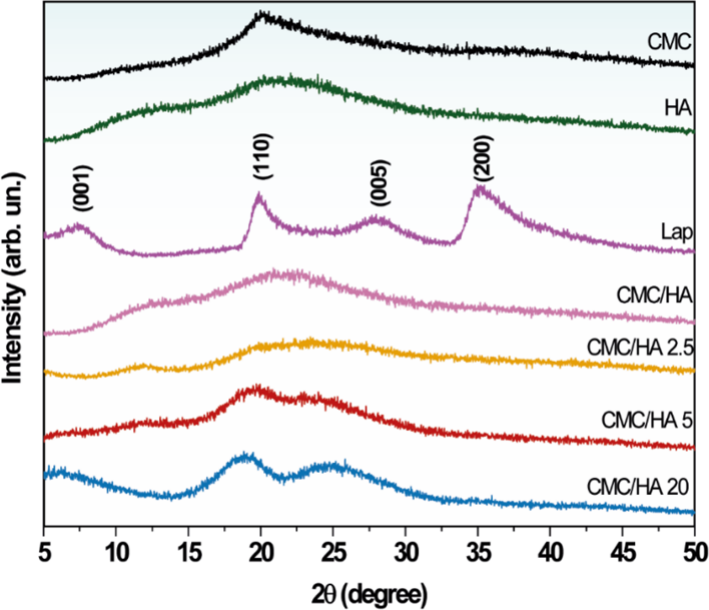
XRD patterns of CMC, HA, Lap powder, and all prepared nanocomposite films.

Figure 2 shows the XRD patterns for CMC, HA, Laponite, and all prepared nanocomposites containing different amounts of Laponite. The CMC and HA diffractograms showed a typically amorphous structure with 2θ reflections of 20° and 22°, respectively. On the other hand, Laponite indicated defined peaks characteristic of a material with a crystallinity degree, as well as it is possible to notice the basal peak (001) of the lamellar material at 2θ = 7.30° with *d*_001_= 1.21 ± 0.01 nm. In the XRD patterns of CMC/HA 2.5 and CMC/HA 5 nanocomposites, there are no reflections attributed to the Laponite structure, suggesting the formation of an exfoliated nanocomposite.^37^ However, in the case of CMC/HA 20, the reflection of the Laponite basal peak (001) at 5.36° indicates a random dispersion of exfoliated Laponite within the polymeric chains of CMC/HA. This suggests that the polymeric matrix and the lamellar material were partially combined through the exfoliation and/or tactoid formation of Laponite in the polymeric matrix, leading to an increase in the interlayer spacing of *d*_001_= 1.65 ± 0.01 nm. In addition, it can be observed that increased concentrations of Laponite in the polymer matrix promote particle agglomeration and the formation of tactoids, where some Laponite layers are stacked within the nanocomposite.

**Figure 3:**
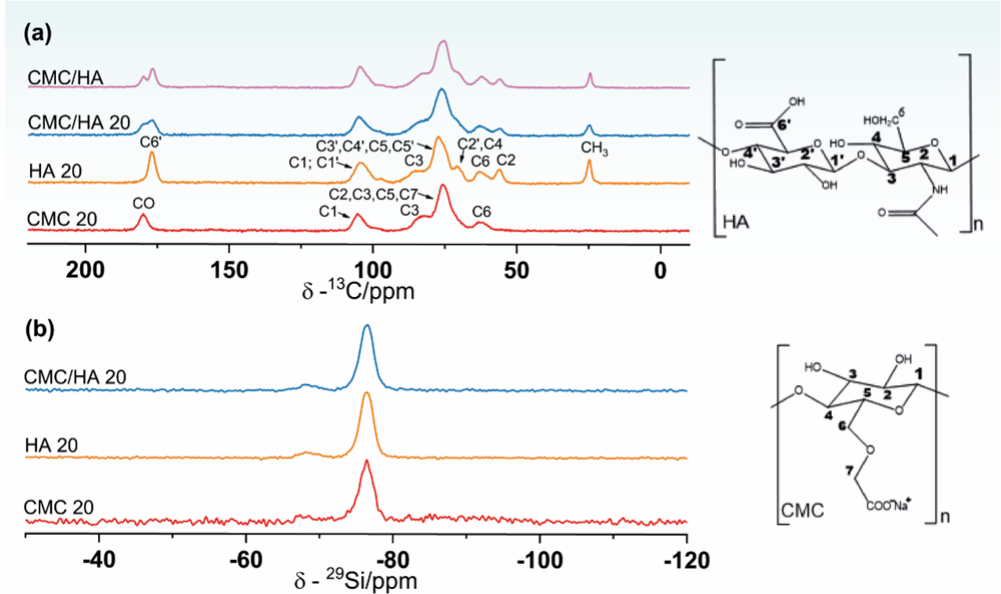
CP-MAS NMR spectra of ^13^C (a) and ^29^Si (b) nuclei for nanocomposite films.

To understand the formation and distribution of Laponite in the nanocomposite films, solid-state ^29^Si and ^13^C NMR was used as a structural probe to the nanocomposites with a higher concentration of Laponite. Additionally, nanocomposite films only with CMC or HA were prepared for this analysis to verify if there was a preference for interaction between the biopolymer and the synthetic clay. From the ^29^Si and ^13^C nuclei chemical environments, it is possible to follow the interactions between nanocomposite films and Laponite added. The ^13^C NMR spectra of CMC, HA, and CMC/HA 20 are shown in Figure 3a. The data for the CMC/HA 20 nanocomposite films show the characteristic peaks of CMC and HA, confirming the success of HA incorporation. The peak at 25 ppm is characteristic of the methyl group of acetamide, and the peak at 56 ppm is relative to the C2 (N-acetylated) of HA. The broad peak observed in the 66 to 95 ppm range corresponds to carbons C-2,3,5 and C4 of the CMC and C2′,4 and C-3′,4′,5,5′ of the HA, respectively. Additionally, the peaks at 101, 176, and 179 ppm correspond to the carbon C1 and the carbonyl peak of HA and CMC, respectively.^38−40^ For ^29^Si NMR data, depicted in Figure 3b, peaks are observed at −76 and −68 ppm, characteristic of the crystalline Laponite,^18^ indicating that the Laponite particles were incorporated in the nanocomposite films. However, the NMR results suggest that there is no chemical interaction between Laponite and CMC/HA, also observed by Perotti et al.^18^ The results agree with those observed data by XRD, and the exfoliated Laponite is randomly dispersed into the polymeric chains of CMC/HA and the polymeric matrix. This observation is further supported by the TG/TDA analysis presented below, which indicates a decrease in the organization of the polymeric chain with increasing Laponite concentration. The observed behavior can be attributed to the random dispersion of Laponite nanoparticles between the polymeric chains, leading to a reduction in the number of cross-interactions among them.

### 3.2 Thermal Analysis

**Figure 4:**
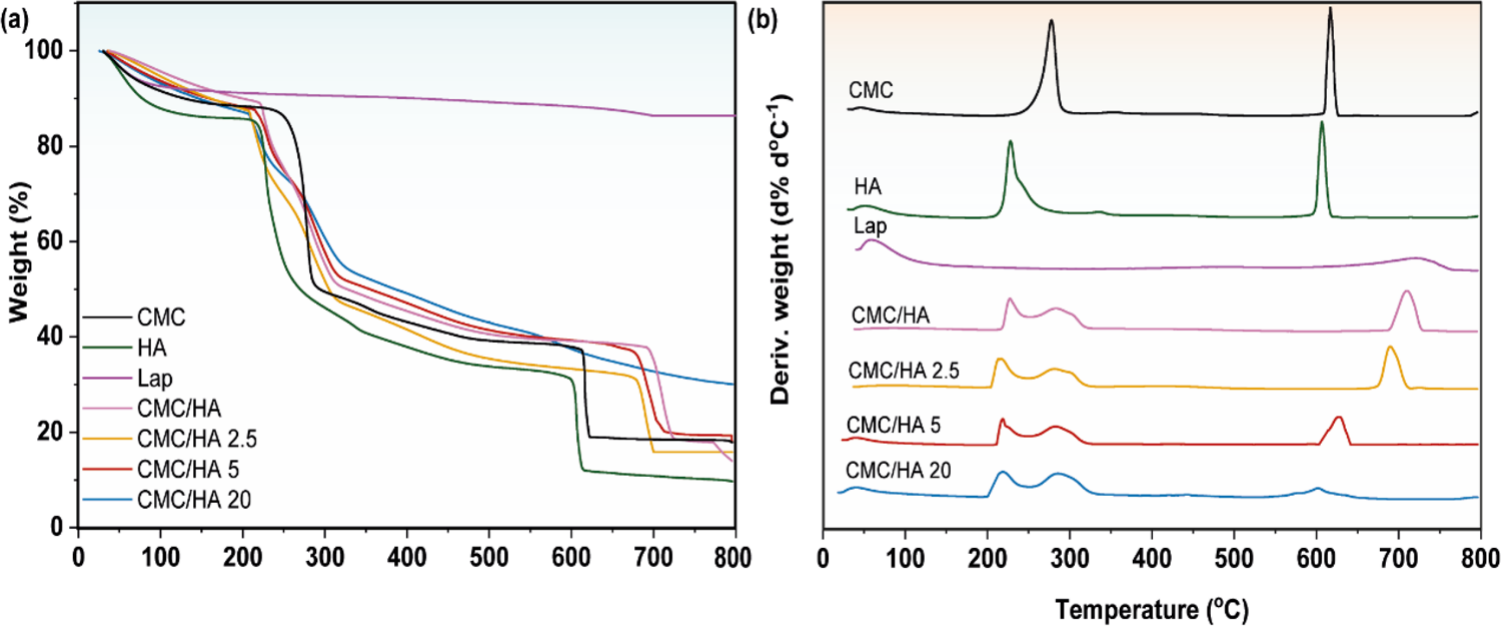
TGA (a) and DTG (b) of CMC, HA, Lap, and nanocomposite films showing the main mass loss profiles.

Figure 4 shows the TGA and derivate thermogravimetric (DTG) curves for CMC, HA, Laponite, and nanocomposites in the presence of different Laponite contents. CMC presented three mass loss processes: the first one was attributed to the water loss between 30 and 100 °C, and the second and third processes at 277.9 and 616.8 °C were attributed to thermal degradation of cellulose from side chain degradation and CO_2_ loss and were responsible for the mass loss of 9.4, 45.6, and 27.1%, respectively.^41,42^ HA showed three mass loss processes, where the first one was attributed to the water loss from the material between 30 and 100 °C. The second and third processes refer to the polysaccharide degradation and HA decomposition at 227.3 and 606.8 °C, respectively.^10,43,44^

The Laponite thermal profile showed two mass loss processes. The first one was attributed to the adsorbed water between 30 and 150 °C with a mass loss of 8.4%. The second process occurred at 692.2 °C, and it is related to the dehydroxylation of the lamellar material layers with a mass loss of 5.2%.

The nanocomposites showed similar mass loss profiles with three mass loss processes. The first and second processes are characteristics, the HA and CMC, which make up the polymer matrix, respectively. In addition, CMC/HA also showed a third mass loss process at 710.1 °C. From Figure 4b, it is possible to notice that the first process for CMC/HA showed a slight reduction in temperature when compared to HA, while the second process showed an increase of 5.7 °C about CMC. The third mass loss process showed a significant increase of 93.3 and 103.3 °C compared to CMC and HA, respectively.

On the other hand, the nanocomposites showed a reduction in temperatures related to the HA component (first mass loss process), reaching reduction values of 11.3, 9.5, and 9.3 °C for CMC/HA 2.5, CMC/HA 5, and CMC/HA 20, respectively. Moreover, the second mass loss process, referring to the CMC component of the polymer matrix, showed an increase in temperature between 4.5 and 8.1 °C. The third loss process also showed a reduction for all treatments concerning CMC/HA, reaching a reduction between 20.4 and 108.4 °C. This thermal behavior suggests a decrease in polymeric chain organization as a function of Laponite concentration and the interaction of Laponite with the polymer matrix, which influenced the polymer crystallinity in some segments. This can be associated with the random dispersion of Laponite nanoparticles between the polymeric chains, which reduces the cross-interactions between them due to the physical interaction of Laponite with the polymers.

Although the films did not present stability at high temperatures, as mentioned in Section 1, the films are still suitable for applications in the biomedical field and integrated chemical sensors, as these applications work at low temperatures of operation, and the precursors are biocompatible.

### 3.3 Stress–Strain Analysis

Mechanical parameters were examined by a uniaxial tensile test (Figure S3). For polymeric films, elastic deformation occurs at sufficient low stress, causing the unfolding and alignment of chains, resulting in reversive changes of intermolecular forces. Accordingly, a small Young’s modulus is expected for substrates with elevated susceptibility to deformation.^45^ As follows, the HA polymer has an ultimate tensile strength (UTS) of 277.2 MPa, an elongation at break (EAB) of 30.9%, and Young’s modulus (YM) of 56.1 ± 1.7 MPa. On the other hand, the pure CMC film has a UTS of 171.0 MPa, an EAB of 9.60%, and a YM of 47.9 ± 1.4 MPa (Table S2). According to the results, the CMC/HA films exhibited lower ultimate tensile strength and Young’s modulus compared to the pure films, indicating reduced resistance to stretching but increased flexibility with the addition of HA to CMC. This observation is consistent with previous studies that have reported higher EAB values for CMC/HA films compared to pure CMC films.^12,25,26^ However, the incorporation of 2.5 and 5% Laponite into the CMC/HA matrix resulted in increased UTS and YM values compared with the CMC/HA films. This indicates that the addition of 2.5 and 5% Laponite enhances the film’s resistance to stretching while still maintaining a certain degree of deformability, as evidenced by the YM values. On the other hand, with the 20% loading of the synthetic clay, the UTS lowered, probably because of the Laponite aggregation, which causes a point of stress concentration, reducing the surface area of interaction between the biopolymers.^46^ Nevertheless, CMC/HA 5 is more resistant to stretch than all the CMC/HA composites including the pure and CMC/HA films with 370.6 MPa UTS, 16.5% EAB, and 51.0 ± 1.2 MPa YM, making it a good candidate for final application as a substrate for OLED devices.^47^

### 3.4 OLED Characterization

**Figure 5:**
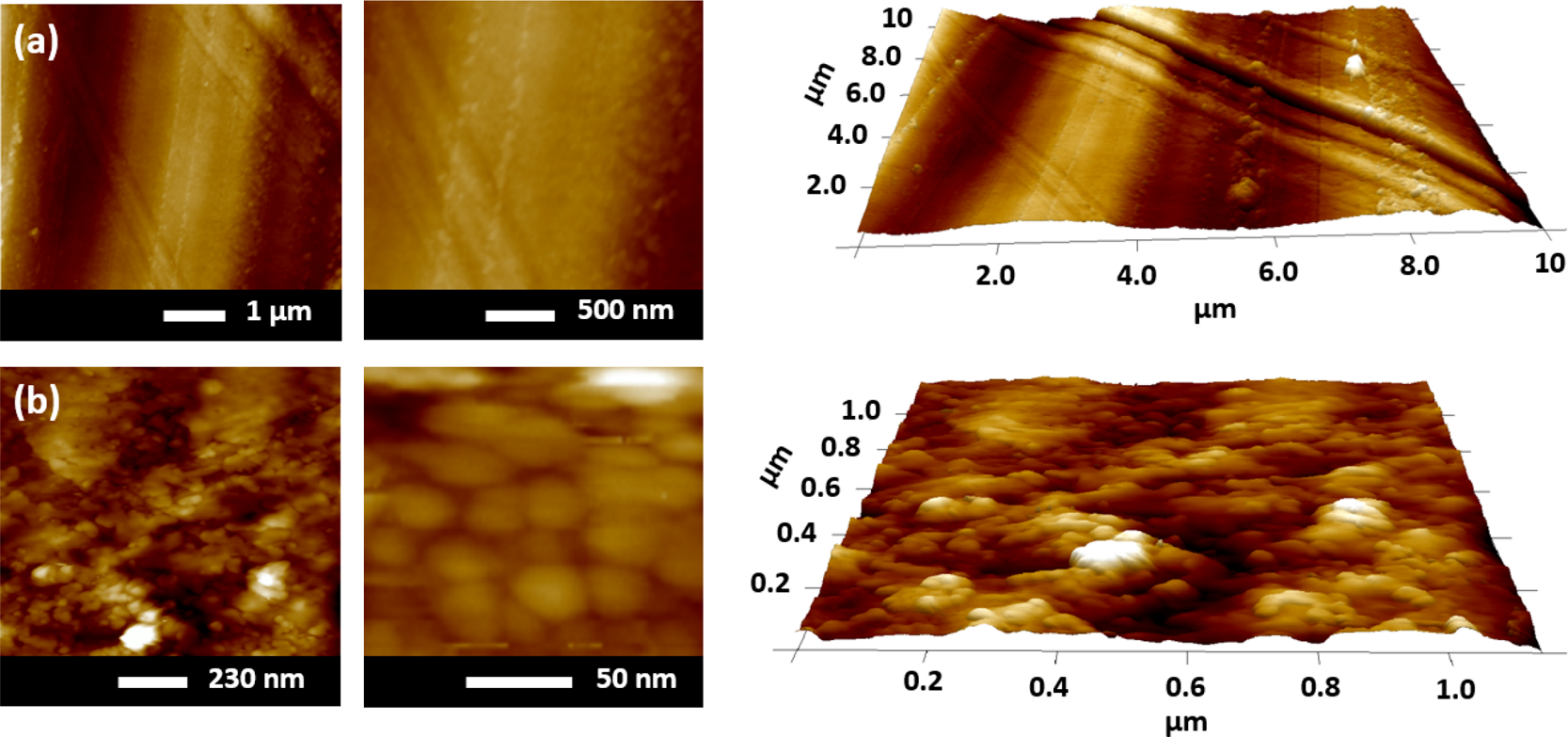
AFM surface images of the pristine CMC/HA 5 (a) and ITO-coated CMC/HA 5 substrates (b) with 2D and 3D visualization.

Atomic force microscopy (AFM) measurements were performed using a Bruker model Multimode-8 operated in peak force mode with a tin-doped silicon cantilever (spring constant *K*_s_ = 3 N/m and resonant frequency of 75 kHz). Figure 5a,b shows the surface morphologies of the pristine CMC/HA 5 and ITO-coated CMC/HA 5 substrates. The pristine CMC/HA 5 substrate (Figure 5a) exhibits a smooth and even surface. However, the presence of aggregated particles can be observed at specific points. This phenomenon is generally explained by the difficulty in achieving perfect uniformity in the filmogenic solution, uneven solvent evaporation, and the aggregation of hyaluronic acid units due to interactions.^44,48,49^ The observed striations can be attributed to the surface of the polystyrene Petri dishes during the solvent casting process.

Figure 5b reveals the presence of ITO grains responsible for the granular appearance observed on the surface of the ITO film using AFM. The ITO film deposited on the CMC/HA 5 substrate showed an electrical resistivity of 5.0 × 10^–4^ Ω/m (sheet resistance of 35 Ω/□), a value very close to that exhibited by the commercial ITO-coated glass substrate.^50^

This granular appearance indicates the crystalline nature of the material. During the sputtering deposition of ITO, the material undergoes rearrangement, resulting in the formation of grains in accordance with the crystallinity standards of ITO. This phenomenon is influenced by factors such as nucleation and grain growth.^50^ It is widely acknowledged that ITO films exhibit a cubic bixbite polycrystalline structure^51^ as confirmed by the diffraction pattern shown in Figure S4, and this polycrystallinity is commonly attributed to the enhanced electrical conductivity compared to amorphous ITO.^52^

**Figure 6:**
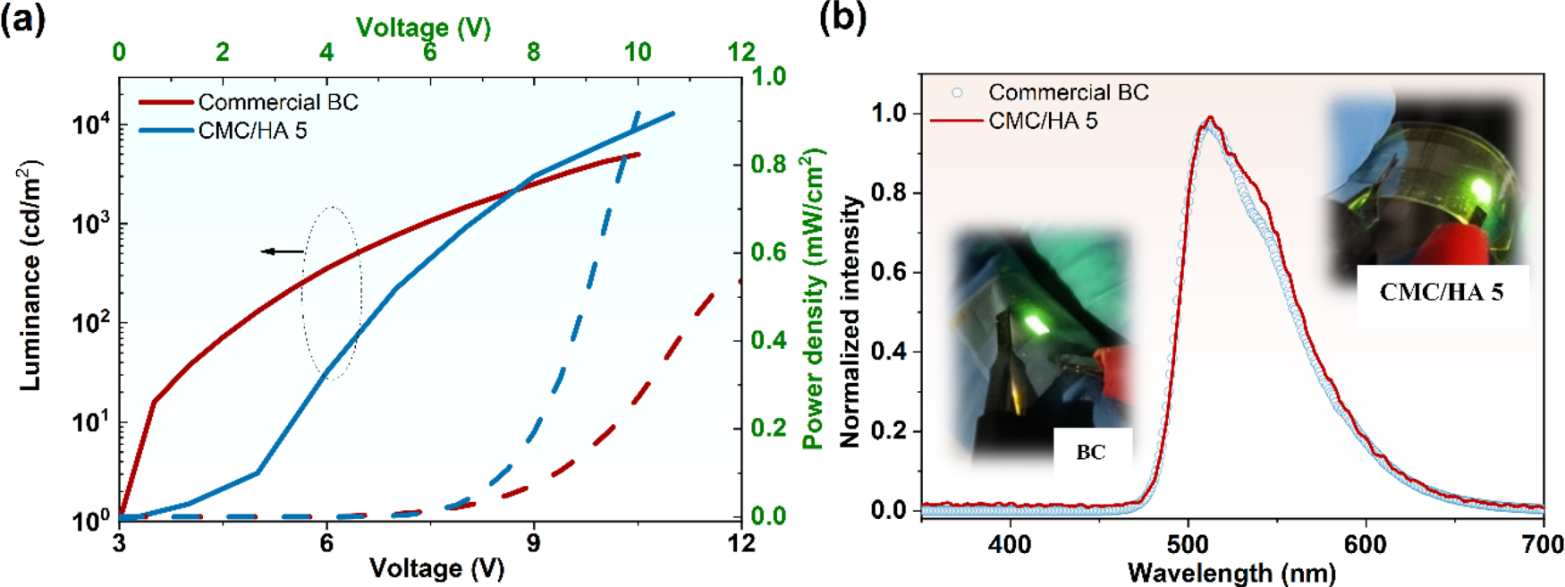
(a) Characteristic curves of OLEDs with commercial BC and CMC/HA 5; solid curves associated with luminance measurements and dashed curves with power density; (b) electroluminescence spectra of OLEDs fabricated onto commercial BC and CMC/HA 5. Inset: pictures of the fabricated devices (photographs taken by the authors).

Considering the well-established literature^53−56^ in the field of OLEDs and structural similarity with the nanocomposite studied, the bacterial cellulose (BC) biopolymer was selected as a reference substrate material for performance comparison with the CMC/HA 5 substrate for the fabrication of conformable OLEDs. Figure 6a shows the characteristic curves for the OLEDs based on commercial BC and CMC/HA 5. The maximum luminance and irradiance values for commercial BC were 5000 cd/m^2^ and 0.5 mW/cm^2^ with a turn-on voltage around 3.0 V, while for CMC/HA 5, these values were 12890 cd/m^2^ and 0.92 mW/cm^2^ for the same turn-on voltage, respectively. Both devices have an active area of about 3 mm^2^ and present Ir(ppy)_3_ characteristic peak emission in their electroluminescence spectrum (Figure 6b). For the OLED fabricated onto the glass/ITO substrate, the values were 72000 cd/m^2^ and 8.4 mW/cm^2^ with EQE_max_ = 8.8%.

**Figure 7:**
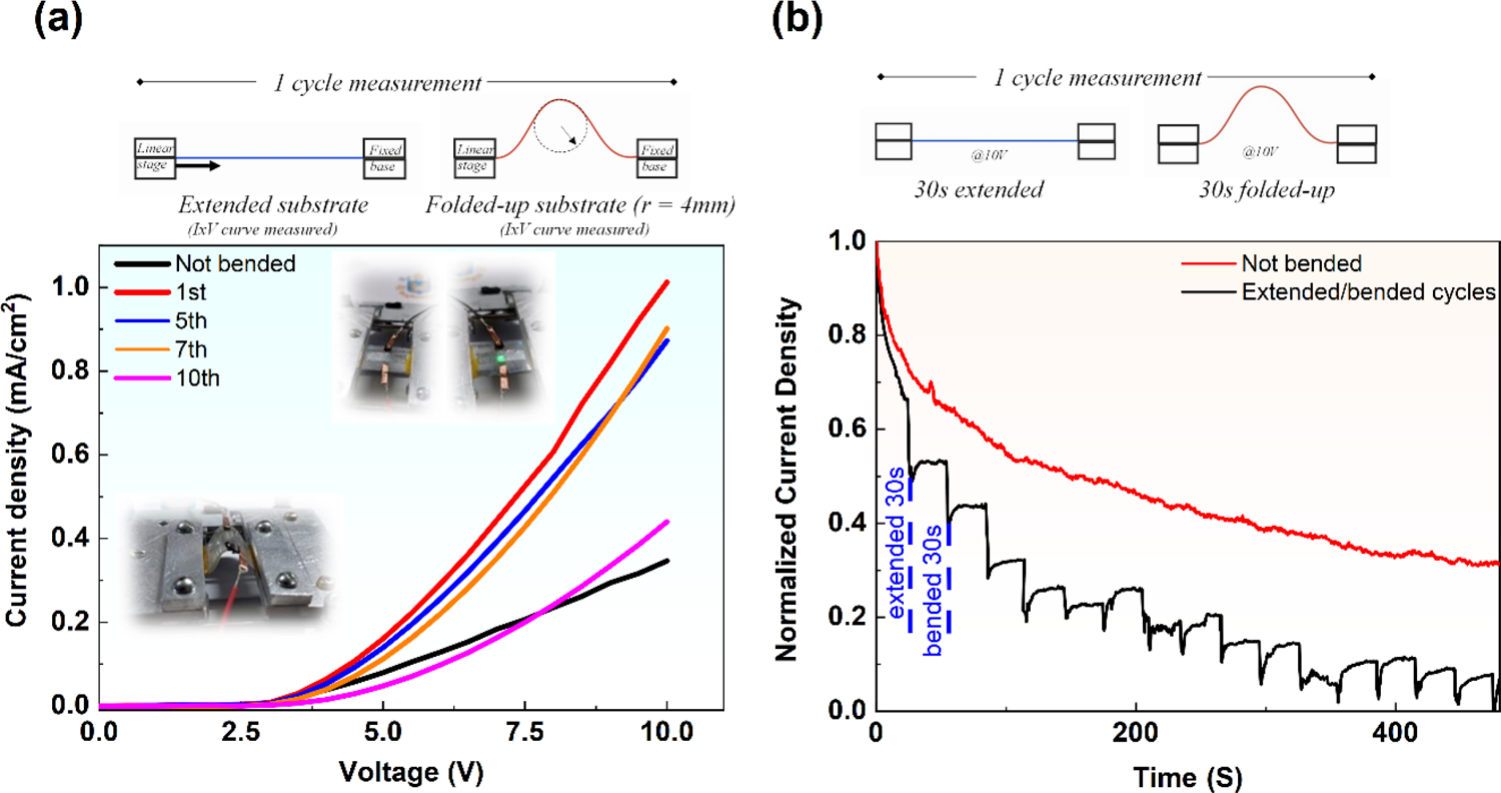
(a) First bending test: current density–voltage curves for 10 cycles of flexibility test. (b) Second bending test: current density values as a function of time for several bending cycles. The red line is the current density drift of a nonbended device (photographs taken by the authors).

Even though the CMC/HA 5-based OLED produces more than twice the luminance and irradiance of the commercial BC-based device, in terms of efficiency, it shows four times less (EQE 2.5% CMC/HA 5 and 10% commercial BC, @1000 cd/m^2^) due to its higher current density. However, it is evident that the overall performance of the CMC/HA 5-based OLED as a conformable and biocompatible substrate is comparable with those based on commercial BC with additional features mentioned previously. To demonstrate the CMC/HA 5 substrate conformability, we performed two different bending tests (Figure 7).^57^ In the first test, we measured *J*–*V* curves for each configuration from 0 to 10 V with a 0.5 V step. The cycle is extended OLED–bended (*r* = 4 mm) OLED. After 10 cycles, the current density became lower than the first sweep. It can indicate that the device electrode (SiO_2_/ITO), after many cycles, starts to experience some losses in the conduction properties due to the bend of the substrate. In the second bending test, 10 V was applied continuously in two configurations: for 30 s, the device was left extended, and then the device was bended (*r* = 4 mm) for an additional 30 s. After some cycles, besides the typical current density drift (Figure 7b, red line), the current density stops to experience a decrease and reach a saturation regime.

## 4 Conclusions

The nanocomposite films based on CMC/HA and Laponite successfully produced by a solvent casting technique presented flexibility and high transmittance in comparison with the films without the synthetic clay. Based on the overall characterization, Laponite was incorporated in the polymeric matrix randomly dispersed between the polymeric chains. Among the samples, CMC/HA 5 demonstrated superior performance for conformable OLEDs, as indicated by the stress–strain analysis, showcasing a high tensile strength of 370.6 MPa and the ability to withstand deformation without breaking. This flexibility enables the OLED device to operate under stretched conditions. Additionally, the AFM results confirmed the successful coating of ITO on the CMC/HA 5 films, enabling the development of an OLED device. The comparative analysis of the fabricated OLEDs with commercial BC and CMC/HA 5 substrates reveals similar characteristics and performance metrics. While the CMC/HA 5-based OLED demonstrated higher maximum luminance (12 kcd/m^2^) and irradiance (0.9 mW/cm^2^) values compared to the commercial BC-based device, its efficiency was compromised by the higher current density. Overall, the study highlights the potential of CMC/HA 5 as a viable substrate option with comparable performance to commercial BC and additional conformability features, offering additional advantageous features for future biocompatible OLED advancements.

## Supporting Information Available

Energy diagram of fabricated OLED (Figure S1); ATR-FTIR spectra of CMC, HA, Laponite (Lap) powder, and nanocomposite films (Figure S2); process of mass loss with respective temperature and the amount of Laponite calculated by TGA and DTG curves (Table S1); stress–strain curves for the films (Figure S3); tensile strength, elongation at break, and Young’s modulus of CMC, HA, and nanocomposite films (Table S2); X-ray diffraction pattern of the ITO-coated CMC/HA 5 substrate (Figure S4) (PDF)
