# Supplementary material for: Laponite-Modified Biopolymers as a Conformable Substrate for Optoelectronic Devices
Source: ACS Omega. 2024 Jul 9;9(29):31855–63. doi: 10.1021/acsomega.4c03463 (PMC11270560; doi:10.1021/acsomega.4c03463)
Supplement: Supplementary file 1 — ao4c03463_si_001.pdf [file ao4c03463_si_001.pdf]

# Supporting information

# Laponite-modified biopolymers as a conformable substrate for optoelectronic devices

Bruno S. D. Onishi[a], Rafael S. Carvalho[b], Ricardo Bortoletto-Santos[c], Silvia H. Santagneli[a], Arthur R. J. Barreto[b], Aline M. Santos[b], Marco Cremona[b], Omar G. Pandoli[d,e], Mario N. B. Junior[e], Thales A. Faraco[f], Hernane S. Barud[g], Renan L. de Farias[d], Sidney J. L. Ribeiro[a*] Cristiano Legnani[f*]

[a] *Institute of Chemistry, São Paulo State University (UNESP), 14800-060 Araraquara-SP, Brazil*

[b] *Departamento de Física, Pontifícia Univ. Católica do Rio de Janeiro (PUC-Rio), Rio de Janeiro, Brazil 22451-900*

[c] *Postgraduate Program in Environmental Technology, University of Ribeirão Preto (UNAERP), Ribeirão Preto, Brazil*

[d] *Departamento de Química, Pontifícia Univ. Católica do Rio de Janeiro (PUC-Rio), Rio de Janeiro, Brazil 22451-900*

[e] *Departamento de Engenharia Química e de Materiais, Pontifícia Univ. Católica do Rio de Janeiro (PUC-Rio), Rio de Janeiro, Brazil 22451-900*

[f] *Departamento de Física, Laboratório de Eletrônica Orgânica (LEO), Univ. Federal de Juiz de Fora (UFJF), Juiz de Fora, Brazil 36036-330*

[g] *Univ. de Araraquara (UNIARA), Laboratório de biopolímeros e Biomateriais (BIOPOLMAt), Araraquara, Brazil 14801-340*

## *Corresponding Authors

E-mail: sidney.jl.ribeiro@unesp.br; cristiano.legnani@ufjf.br

Bruno S. D. Onishi and Rafael S. Carvalho are co-first authors with equal contributions and importance

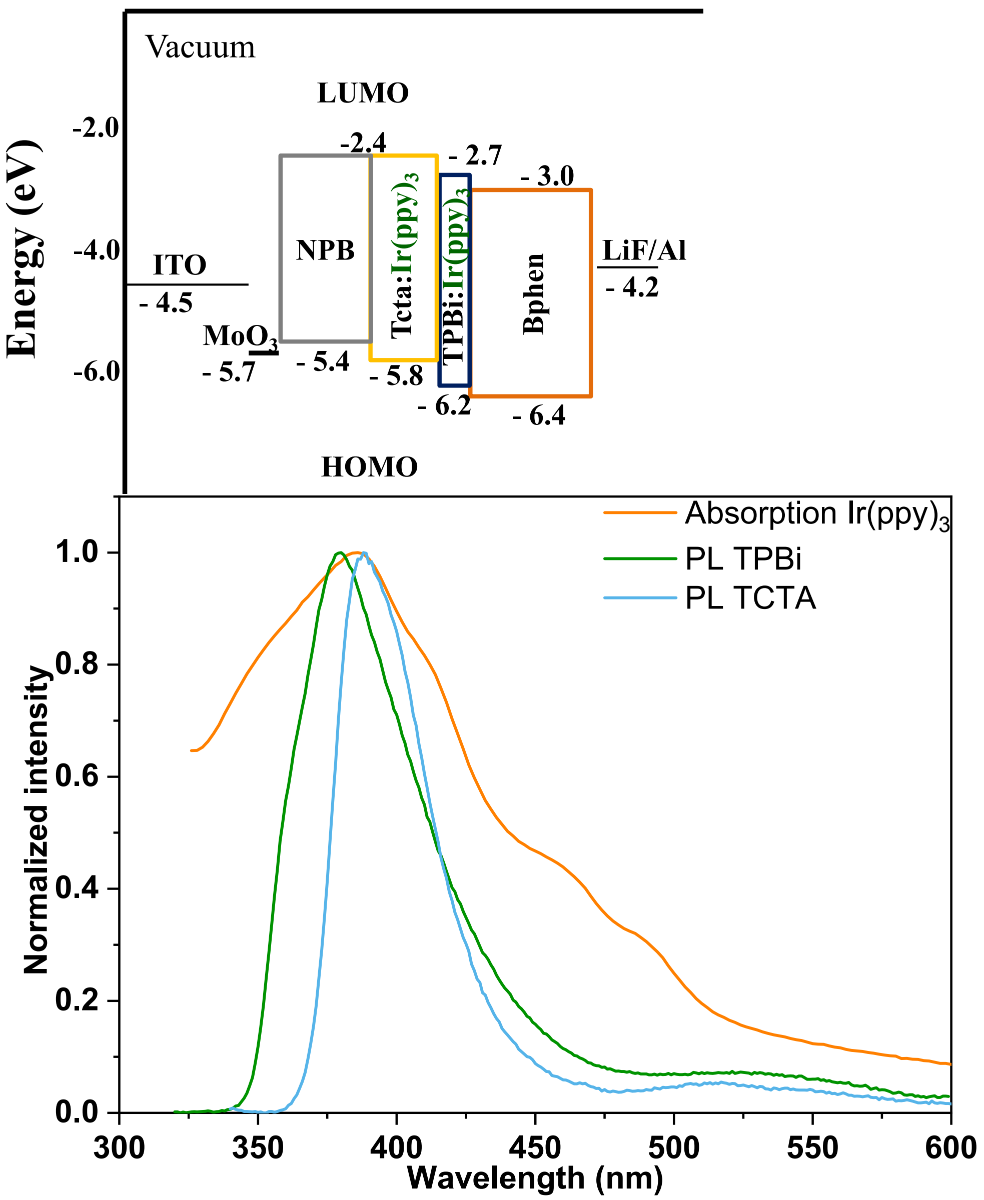


**Figure S1.** Energy level diagram of fabricated OLED (upper) and overlap of the photoluminescence (PL) spectrum of the matrix and absorption spectrum of Ir(ppy)3 in the film solid-state (below) respectively

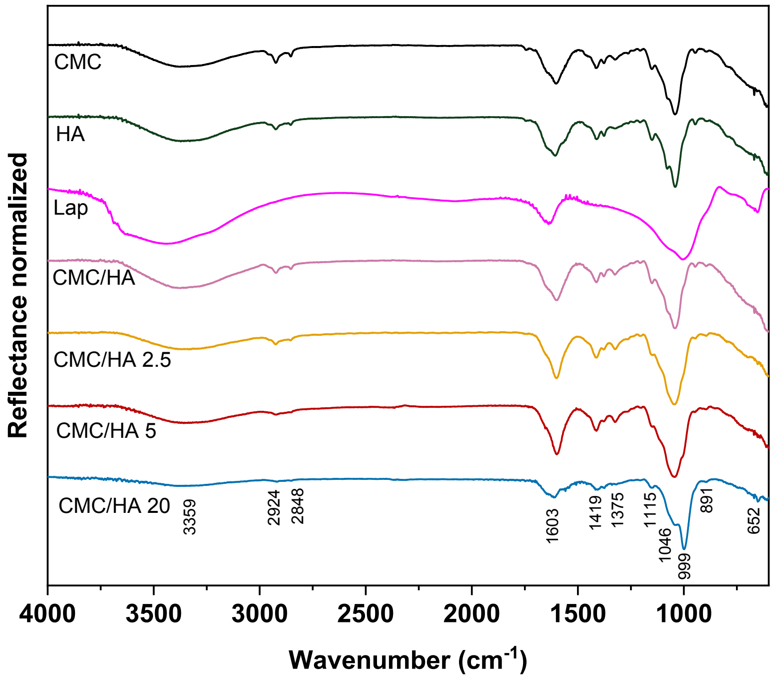


**Figure S2**. ATR-FTIR spectra of CMC, HA, Laponite (Lap) powder and nanocomposites films

**Table S1** - Process of mass loss with respective temperature and the amount of Laponite calculated by TGA and DTG curves

| Treatments | Maximum temperature of mass loss processes (°C) | | | TGA residue (%) | Calculated Laponite (%) or (g) | |
|---|---|---|---|---|---|---|
| **CMC** | - | 277.6 | 616.8 | 17.87 | - | - |
| **HA** | 227.9 | - | 606.8 | 9.67 | - | - |
| **CMC/HA** | 227.3 | 283.3 | 710.1 | 13.77 | - | - |
| **CMC/HA 2.5** | 216.6 | 283.2 | 689.7 | 15.82 | 2.39 | 0.025 |
| **CMC/HA 5** | 218.4 | 282.1 | 627.5 | 18.11 | 5.0 | 0.052 |
| **CMC/HA 20** | 218.6 | 285.7 | 601.7 | 30.07 | 19.05 | 0.240 |

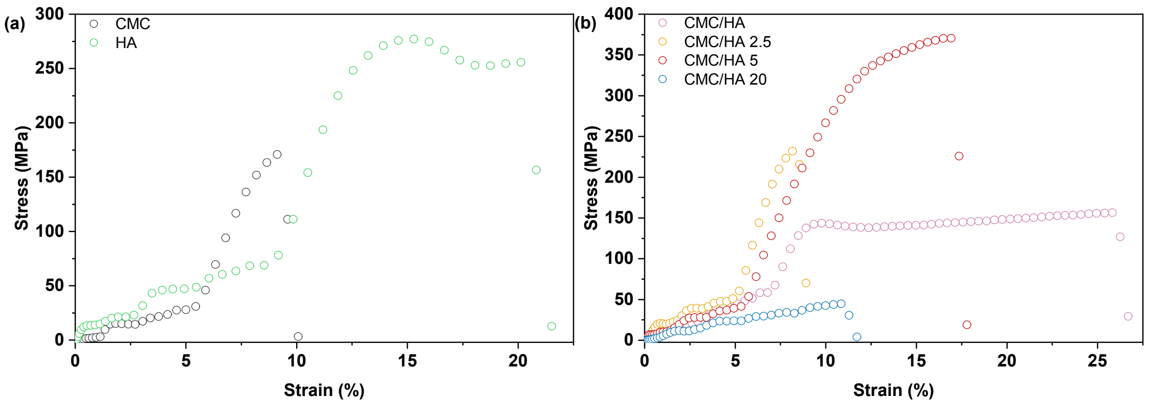


**Figure S3.** Stress-strain curves for the CMC, HA films (a) and CMC/HA, CMC/HA 2.5, CMC/HA 5, and CMC/HA 20 films (b). Experimental conditions: load cell of 50 kgf and crosshead rate 0.83 mm $s^{-1}$, at room temperature.

**Table S2** - Tensile strength, elongation at break and Young's modulus of CMC, HA, and nanocomposites films

| Specimen | Tensile Strength (MPa) | Elongation (%) | Young's modulus (MPa) |
|---|---|---|---|
| **CMC** | 171.0 | 9.60 | 47.9 ± 1.4 |
| **HA** | 277.2 | 30.9 | 56.1. ± 1.7 |
| **CMC/HA** | 143.7 | 25.8 | 40.26 ± 2.7 |
| **CMC/HA 2.5** | 232.0 | 7.80 | 69.6. ± 2.4 |
| **CMC/HA 5** | 370.6 | 16.5 | 51.0 ± 1.2 |
| **CMC/HA 20** | 44.80 | 10.4 | 40.3± 2.7 |

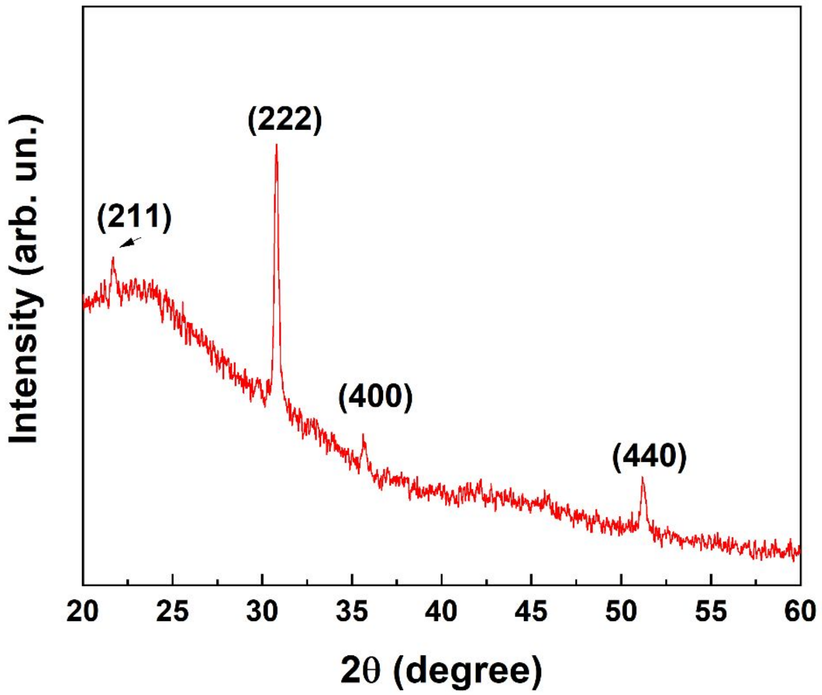


**Figure S4.** X-ray diffraction pattern of the ITO-coated CMC/HA 5 substrate reveals crystal planes corresponding to (211), (222), (400), and (440).
